# Effect of indoor house dust mite concentration on canine atopic dermatitis

**DOI:** 10.3389/fvets.2023.1078306

**Published:** 2023-02-02

**Authors:** Jihee Kim, Ji-Hye Lee, Yunji Song, Ha-Jung Kim

**Affiliations:** ^1^Department of Internal Medicine, College of Veterinary Medicine, Chonnam National University, Gwangju, Republic of Korea; ^2^BK 21 Project Team, College of Veterinary Medicine, Chonnam National University, Gwangju, Republic of Korea

**Keywords:** atopic dermatitis, house dust mites, dog, environment, transepidermal water loss

## Abstract

**Introduction:**

House dust mites (HDM) are regarded as essential environmental allergens not only in human, but also in canine atopic dermatitis (CAD), however, there are only a few studies on the influence of indoor HDM concentration on the disease.

**Methods:**

Our study analyzed the correlation between the indoor HDM concentration, the severity of CAD, and the residential environments in client-owned 35 AD and 13 healthy dogs. We measured the extent of CAD and severity index-04 (CADESI-04), pruritus visual analog scale (PVAS), and transepidermal water loss (TEWL), indoor relative humidity (RH) and analyzed the residential environment questionnaires to evaluate AD severity.

**Results:**

The *Der f 1* concentration had an inverse association with TEWL, and no association with CADESI-04 and PVAS. The *Der f 1* concentration was significantly high in the group living near the green area and 40% or higher RH.

**Discussion:**

Our results suggest two possibilities: (1) Living around green areas and maintaining an appropriate indoor climate may help to improve CAD clinical symptoms. (2) The HDM may contain endotoxin and when present in high concentrations in CAD, they play a preventive role by enhancing the skin barrier function. Further studies with a larger number of dogs may help further elucidate an association between CAD and *Der f 1*.

## 1. Introduction

Atopic dermatitis (AD) is a hereditary pruritic skin disease closely associated with an environmental allergen, and is reported to be related to the house dust mite (HDM) in both dogs and humans (1). Previous studies have shown that the sensitization rate of HDM in the human atopic patients is about 35%, and about 72 and 25% of the canine atopic patients had a positive reaction to *Dermatophagoides farinae* (*D*. *farina*) and *Dermatophagoides pteronyssinus* (*D*. *pteronyssinus*), respectively (1, 2). HDM is affected significantly by the geographic characteristics associated with its ecological property. *D. farinae* is the dominant species of HDM in Korea, followed by *D. pteronyssinus* (3). Several studies have investigated how exposure to HDM in humans becomes a risk factor for asthma or AD through mesuring *Der f 1* and/or *Der p 1* allergen (4–7). In particular, exposure to allergen influences the development of asthma symptoms greatly (8). Although there are fewer studies on the effects of HDM on AD than allergic rhinitis or asthma, there are reports that suggested that high exposure to HDM may worsen AD severity (9, 10). However, despite the higher sensitization ratio to HDM in dogs than in humans (2), there are only a few studies on the association between indoor HDM concentration and CAD, with the previous research failing to reveal any significant correlation (11).

AD is a type 1 hypersensitivity reaction emanating from exposure to environmental allergens such as HDM through an impaired epidermis, resulting in the production of specific IgE (12, 13). The skin barrier is considered to be disturbed in AD skin (14). Transdermal epithelial water loss (TEWL) is an indirect skin barrier function test that evaluates the degree to which water passes from inside to outside in the upper layer of the epidermis (14). Previous studies demonstrated that TEWL was significantly low after treatment in CAD (15), and a positive correlation between TEWL and severity of skin barrier dysfunction had been reported (14, 16).

Korea has a high proportion of people living in apartments with their dogs. Due to space limitations, the number of outdoor activities is very limited; consequently, many dogs spend most of their time indoors. Indoor AD in dogs seems to be very closely related to the living environment in Korea. A better understanding of the relationship between exposures to HDM, the most important of indoor environmental allergens in AD, can help prevent skin symptoms from aggravating in CAD. Therefore, our study aimed to establish a comprehensive long-term strategy for AD treatment by exploring the association between dog residential environment, indoor HDM level, and AD severity. To the best of our knowledge, this is the first study to compare and analyze the indoor HDM concentration with dogs' residential environment and the clinical severity of AD.

## 2. Materials and methods

### 2.1. Study population and exclusion criteria

All animal studies were reviewed and approved by both of the Animal Ethics Committee of Chonnam National University and the Institutional Review Board of Chonnam National University (authorization no. 2021–163 and 1040198-220228-HR-017-01). And the studies were performed in accordance with the guidelines and regulations for the Ministry of Food and Drug Safety of South Korea. Client-owned 35 dogs with AD and 13 healthy dogs without AD were included in this study. All the 13 healthy dogs of the control group and 35 dogs of the CAD group visited the teaching veterinary hospital for dermatological referral or physical examination. CAD was diagnosed based on Favrot's criteria (17). Before participating, written informed consents were obtained from the owners.

Skin scraping was performed for all dogs to exclude dogs that had parasitic infections; dogs that had systemic glucocorticoids applied within 4 weeks prior to the clinical evaluation were ruled out because the medication used could affect their AD symptoms. Dogs that lived outdoors and spent more than two-thirds of their day outdoors were also excluded from this study.

### 2.2. Survey for residential environments

Residential environments were assessed by questionnaires that were completed by dog owners. All methods were performed in accordance with relevant guidelines and regulations. The questionnaire consisted of 10 questions, which were later used for analysis. The questions were as follows: (i) What is your housing type? (ii) What are the surroundings near your residence? (iii) How many times do you ventilate the house a day? (v) Have you moved recently?

### 2.3. Assessment of the severity of AD and epidermal skin barrier function

In the CAD group, the severity of AD was evaluated through Canine atomic durability extents and Severity Index-04 (CADESI-04), and Pruritus visual analog scale (PVAS). CADESI-04 and PVAS were evaluated in the same way as in previously reported literature (18, 19). The evaluation sites of CADESI-04 included (i) ear pinnae (ii) forefeet (iii) axillae (iv) flexural areas (v) ventral tail (vi) inguinal area and (vii) flanks. Skin lesions (erythema, lichenification, and alopecia/excoriation) for each site were scored as none (score 0), mild (score 1), moderate (score 2), and severe (score 3) and evaluated as a total score (18). The clients assessed their dogs' PVAS by the evaluation table consisting of 0 points (Not ichy) to 10 points (Extremely itchy) (19). TEWL was measured in all dogs by GPSkin Barrier^®^ (GPOWER Inc, Seoul, South Korea) devices of the closed chamber system. All the TEWL values were measured after stabilizing for 30 min in a room with temperatures ranging between 24 and 26°C and RH of between 40 and 60%. The average of the values repeatedly measured three times in three anatomical sites, pinna, axial, and inguinal were recorded (20). Those sites were selected based on previous studies (20, 21).

### 2.4. Dust sampling and HDM allergen evaluation

Dust samples were collected using a standard household vacuum cleaner equipped with a 10 um filter paper. The living room and the beddings of the dogs were vacuumed 1 x 1 m^2^ for 2 min. The collected dust samples were sealed in a plastic bag and stored at −20°C until they were analyzed. For extract preparation, 2.0 mL PBS-T (0.05% Tween 20 in phosphate-buffered saline, pH 7.4) was added to 500 mg of the fine dust. After stirring at room temperature for 2 h, they were centrifuged at 2,500 rpm for 20 min at 4°C, removed volume supernatant, and stored at 20°C. The levels of *Der f 1* were measured through ELISA 2.0 kit (Indoor Biotechnologies, Charlottesville VA, USA) for measuring *D. farinae* allergen and *Der p 1* (Indoor Biotechnologies, Charlottesville VA, USA) for measuring *D. pteronyssinus* allergen.

### 2.5. Measurement of temperature and RH

The indoor temperature and RH for 26 CAD dogs and 11 control dogs were measured by an indoor air quality monitoring station (AirGuard K^®^, Kweatherco. Ltd, Seoul, South Korea). The average value measured for 48 h from the date of dust collection and clinical evaluation was recorded. The group above 40%, which is known as an ideal RH for HDM to survive for prolonged periods, was classified as a high RH group and < 40% as a low RH group (22).

### 2.6. Statistical analysis

All values were expressed as mean and standard deviation and preceded normality evaluation. Independence was tested among the survey data through Fisher's exact test for housing type, surroundings, and recent moving, and test for daily ventilation times was done through chi-square. The Shapiro-Wilk tests were conducted to test the normality of all measurement data, but the normality was not satisfied. Therefore, we used the Mann-Whitney U test and Krustal-Wallis test for testing differences between means. Multiple comparisons were conducted followed by Krustal-Wallis test. Spearman test was performed to analyze the correlation between *Der f 1* concentration and AD severity index. For all analyses, statistical significance was determined at a value of *P* < 0.05. Every statistical test was performed with statistical software (Version 9.0; GraphPad Prism, Inc., La Jolla, CA).

## 3. Results

### 3.1. Patient's epidemiological characteristics

The study included the CAD group consisting of 35 dogs with atopic dermatitis and the control group consisting of 13 healthy dogs. The epidemiologic characteristics of each group are summarized in Table 1. The CAD group consisted of 16 males, which were all castrated (45.7%) and 19 females [5 intact (14.3%), and 14 spayed (40.0%)]. Based on the age at the time of analysis, the average age of the CAD group was 7.23 ± 3.52 years old (range 1–15 years). Maltese was the primary breed (*n* = 12, 34.3%), followed by Bichon Frise (*n* = 5, 14.3%), Poodle (*n* = 4, 11.4%), and American Cocker Spaniel (*n* = 3, 8.6%). And Pomeranian, Shi-tzu and Mixed breed were followed (*n* = 2, 5.7% each). Dachshunds, French Bulldog, Boston Terrier, Chihuahua and Golden Retriever were also included (*n* = 1, 2.8% each). The control group consists of an intact male (7.7%), 6 castrated males (46.2%) and 6 spayed females (40.0%). The average age was 5.84 ± 3.57 years old (range 2–13). Mixed breed and Poodle were mostly included (*n* = 4, 30.8% each), followed by Maltese (*n* = 3, 23.7%), Chihuahua, and Miniature pincher (*n*= 1, 7.7% each).

**Table 1:** Epidemiologic characteristics of 35 AD dogs and 13 healthy dogs.

**Signalment**		**AD dogs (*****n*** = **35)**		**Healthy dogs (*****n*** = **13)**	
		***n***	**%**	***n***	**%**
**Sex**					
	Intact male	0	0.0	1	7.7
	Castrated male	16	45.7	6	46.2
	Intact female	5	14.3	0	0.0
	Spayed female	14	40.0	6	46.2
**Age**					
	< 4 years	5	14.3	5	38.5
	4–7 years	11	31.4	3	23.0
	>7 years	19	54.3	5	38.5
**Breed**					
	Maltese	12	34.3	3	23.7
	Bichon frise	5	14.3	0	0
	Poodle	4	11.4	4	30.8
	American cocker spaniel	3	8.6	0	0
	Pomeranian	2	5.7	0	0
	Shi-tzu	2	5.7		
	Mixed	2	5.7	4	30.8
	Others^a^	5	14.3	2	15.3

a Dachshunds, French Bulldog, Boston Terrier, Chihuahua, Golden Retriever, Miniature pinscher.

### 3.2. Residential survey results

**Figure 1:**
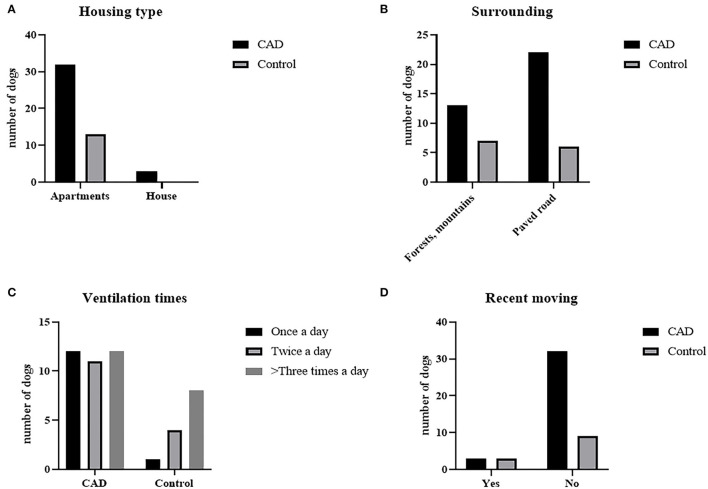
Residential environmental survey results of CAD (*n* = 35) and control groups (*n* = 13). **(A)** Housing type, **(B)** surrounding environment, **(C)** daily ventilation times, and **(D)** whether they moved recently.

The results are graphitized in Figure 1. On the question of the housing type, 91.4% of the CAD group (*n* = 32) and all control groups (*n* = 13) answered that they lived in apartments (*P* = 0.33). 37.14% (*n* = 13) of the CAD group and 53.9% (*n* = 7) of the control group answered that there were mountains or forests near their houses, while others answered that there were paved roads and housing complexes near their houses (*P* = 0.17). For the ventilation times, 34.3% (*n* = 12), 31.4% (*n* = 11), 34.3% (*n* = 12) of the CAD group answered once a day, twice a day, and more than three times a day, respectively. 2% (*n* = 1), 8.3% (*n* = 4), and 50% (*n* = 8) of the control group answered that they ventilated once, twice, and more than three times a day, respectively (*P* = 0.1242). 6.8 % of the CAD and the control group (*n* = 3, each) moved to a new house recently (*P* = 0.19).

**Figure 2:**
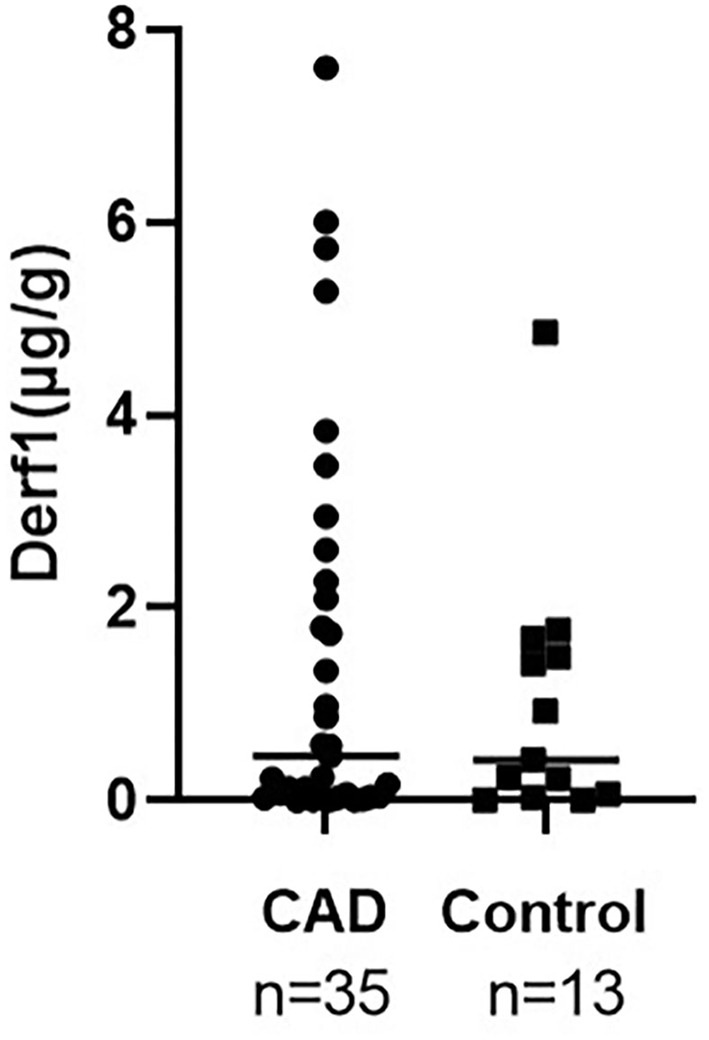
Comparison of indoor *Der f 1* concentration between CAD and control groups. It was higher in CAD without statistical significance (CAD; 1.472 ± 2.04 μg/g; *n* = 35, control; 1.014 ± 1.342 μg/g; *n* = 13, *P* = 0.4).

Correlation between indoor HDM concentration, residential survey, AD severity index and indoor climates The *Der f 1* and *Der p 1* concentrations in the dust samples were measured for all cases. *Der p 1* was not detected in most samples, so it was excluded from this study. *Der f 1* concentrations for CAD group and control group were not statistically significant, but the mean value was higher in CAD groups (CAD; 1.472±2.04 μg/g; *n* = 35, control; 1.014 ± 1.342 μg/g; *n* = 13, *P* = 0.4). The results were graphited in Figure 2.

**Figure 3:**
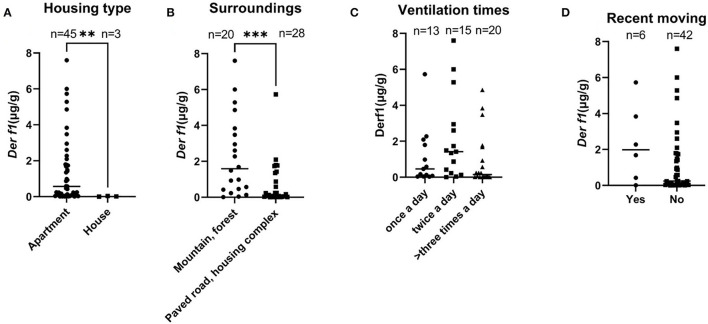
Graphs showing differences in *Der f 1* concentration according to answers of the survey for the 48 dogs (^**^P < 0.01, ^***^P < 0.001). **(A)** Housing type, **(B)** surrounding environment, **(C)** daily ventilation times, and **(D)** whether they moved recently.

Table 2 summarizes the data comparing the residential environmental survey analysis with the *Der f 1* concentration. There was a significant difference in *Der f* 1 concentration according to the responses on the housing type and the surroundings in the CAD group. In the Housing type, the group who answered that they lived in an apartment showed a higher *Der f 1* concentration than the detached house (total; ^**^P = 0.0031, CAD; ^**^P = 0.0035). All the dogs in control groups answered that they lived in apartments thus they were excluded from the statistical analysis. The *Der f 1* concentration was significantly higher in the group that answered that there were mountains or forests around their houses than in the group that did not (total; ^***^P = 0.0006, CAD; ^**^P = 0.0011). In the control group, the dogs who were living near mountains or forests had a higher *Der f 1* concentration, but it was not statistically significant (*P* = 0.1474). There was no association in *Der f 1* with the daily ventilating times and recent moving. These results are graphited in Figure 3.

**Table 2:** The association of indoor *Der f 1* concentration (μg/g) with residential survey.

**Question**	**Answer (Mean ±SD)**	**Total (*N* = 48)**	**CAD (*n* = 35)**	**Control (*n* = 13)**
Housing type	House	0.015 ± 0.018	0.015 ± 0.018	N/A
	Apartments	1.437 ± 1.904	1.608 ± 2.084	1.014 ± 1.342
	*P*-value	^**^0.0031	^**^0.0035	
Surrounding	Mountains, forests	2.293 ± 2.241	2.784 ± 2.416	1.379 ± 1.655
	Paved road, housing complex	0.6732 ± 1.203	0.696 ± 1.307	0.589 ± 0.795
	*P*-value	^***^0.0006	^**^0.0011	0.1474
Ventilation times	Once a day	1.110 ± 1.613	1.199 ± 1.650	0.038 ± 0.0
	Twice a day	2.143 ± 2.368	2.598 ± 2.620	0.8919 ± 0.6593
	≥Three times a day	0.9067 ± 1.474	0.7127 ± 1.386	1.198 ± 1.649
	*P*-value	0.0712	0.0972	0.6424
Recent moving	Yes	2.327 ± 2.156	3.948 ± 1.727	0.707 ± 0.872
	No	1.208 ± 1.817	1.240 ± 13.93	1.107 ± 1.48
	*P*-value	0.0899	^*^0.0127	0.4585

* P < 0.05,

** P < 0.01,

*** P < 0.001.

**Figure 4:**
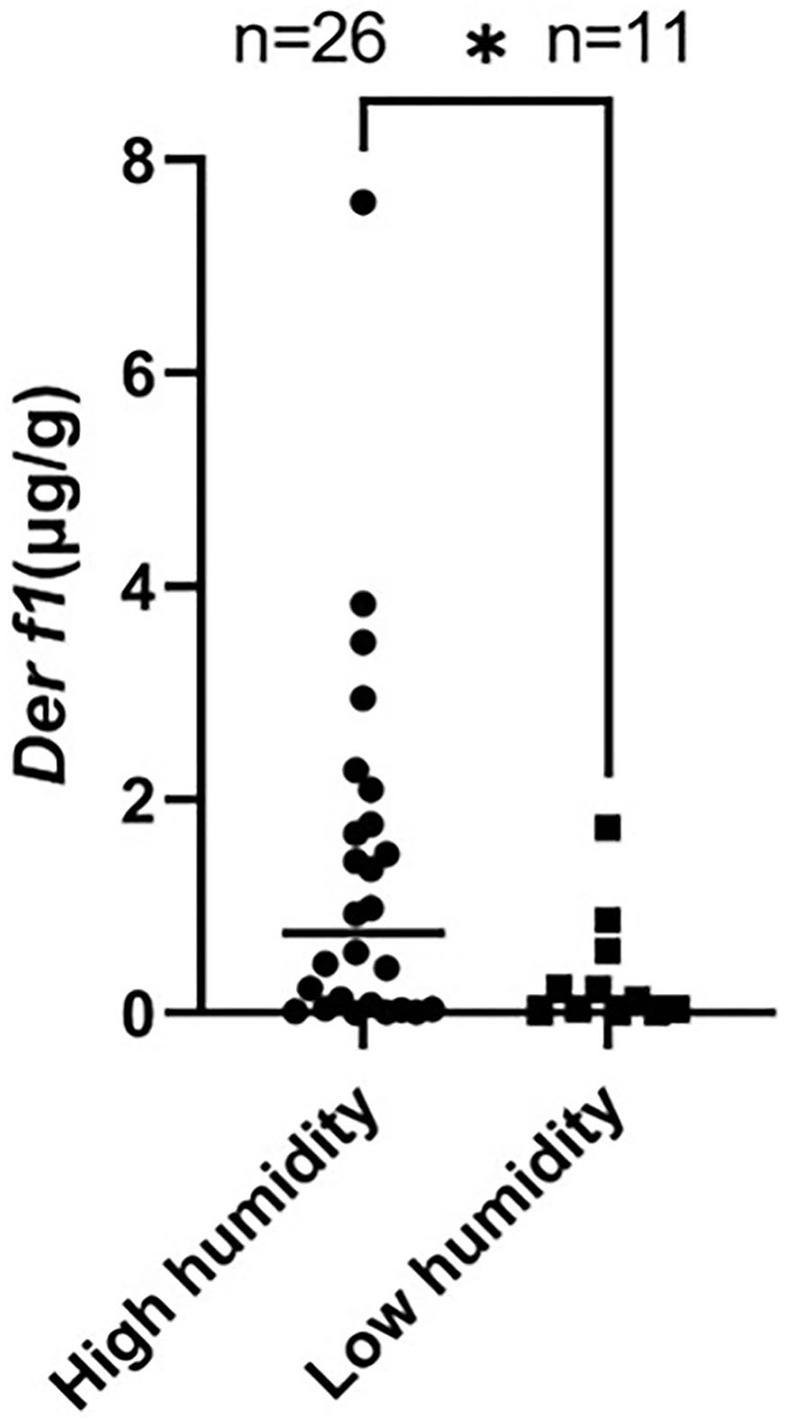
Comparison of the *Der f 1* concentration between high RH group (>40%) and low RH group (<40%). The *Der f 1* concentration was significantly high in the high RH group (high RH (*n* = 26): 1.305 ± 1.713 μg/g, low RH (*n* = 11): 0.3534 ± 0.536 μg/g, ^*^P = 0.0349).

**Figure 5:**
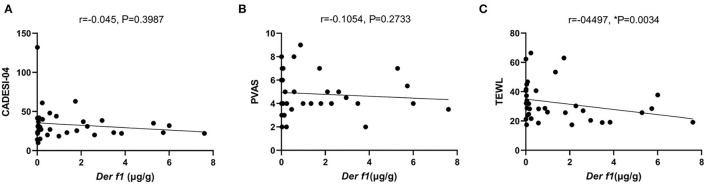
The graph of the correlation *Der f 1* with **(A)** CADESI-04, **(B)** PVAS, and **(C)** TEWL in CAD group (*n* = 35). TEWL (r = −0.3424, ^*^P = 0.02) in the CAD group showed a moderately negative correlation with statistical significance.

Indoor temperature and RH were measured in 26 CAD and 11 control dog houses. The mean indoor temperature for the 37 dogs' houses was 25.99 ± 2.485°C and the RH was 43.06 ± 8.651%. The RH for each group was compared in the housing type and surroundings, where the *Der f 1* concentration was significantly different in the residential survey. In the Housing type, there was no significant difference in the RH between the group living in apartments (*n* = 34) and the group living in detached houses (*n* = 3) (apartment: 43.48 ± 8.681%, detached house: 38.30 ± 8.163%, *P* = 0.1725). The RH was significantly higher in the group living near mountains (*n* = 16) than others (*n* = 21) (mountain: 46.82 ± 6.518, paved road: 40.20 ± 9.107, ^**^P = 0.0094). A high RH group (*n* = 26) and a low RH group (*n* = 11) were divided based on 40%, the *Der f 1* concentration was significantly high in the high humidity group (high RH: 1.305 ± 1.713 μg/g, low RH: 0.3534 ± 0.536 μg/g, ^*^P = 0.0349). The results were showed in Figure 4. Analysis was performed using the Spearman correlation coefficient between *Der f 1* and CADESI-04, PVAS, and TEWL in the CAD group (*n* = 35). CADESI-04 (r = −0.045, *P* = 0.3987), PVAS (r=-0.1054, *P* = 0.2733) had negative correlation with *Der f 1* without statistical significance. TEWL (r = −04497, ^*^P = 0.0034) showed moderate negative correlation with statistical significance. The results were graphitized in Figure 5.

## 4. Discussion

Climatic conditions such as temperature and humidity significantly affect HDM's growth, survival, and allergen production (23, 24). Globally, regional characteristics are associated with HDM fauna, and the sensitization risk for HDM is considerably high in subtropical or tropical regions with high temperature and humidity (24, 25). Korea's climate has the characteristics of the north-east region, similar to the US and Europe, where D. farinae is the dominant species that are resistant to relatively low temperatures and lower humidity than *D. pteronyssinus* (26). Although we measured both *Der f 1* and *Der p 1* allergens in our study, *Der p 1* was not detected in most of the samples, which was considered to be characterized by the geographical area.

HDM is also affected by the urbanization and industrialization of a city (27). As a result of the urban heat island (UHI) effects, urbanization increases general temperature of the atmosphere and decreases in RH (27). In addition, urbanization affects mite biology by causing air pollution and climate change. The type and frequency of HDM sensitized by the elderly and young in places where urbanization progressed rapidly were different in previous studies (27, 28). The analysis comparing the concentration of *Der f 1* with the residential environment was significantly higher in apartments than in detached houses and in the groups with mountains or forests near the houses. However, the number of dogs living in detached houses was small (*n* = 3), and all three dogs were included in the group that answered that there are mountains or forests near the house. Thus, it can be concluded that the geographical characteristics involved in the surroundings rather than the effect of the housing type affected the *Der f 1* concentration in our study.

Creation of urban green areas have been suggested as a way of improving environmental effects caused by urbanization and industrialization. The composition of the green space in the city alleviates the atmospheric particle matter pollution (29). The environment seemed to affect AD. A previous study described that green area is inversely associated with AD in humans (30). Living near the urban green area could reduce exposure to traffic-related air pollution, and consequently, prevent effects that have been reported in infantile AD (31). In addition to alleviating air pollution, the urban green area is known to have cooling and humidifying effects through solar radiation prevention and evapotranspiration (32). Living near the urban green areas has a relatively higher RH and promotes an environment suitable for the growth of HDM. Our study presumed that the *Der f 1* concentration was significantly higher in houses near mountains or forests due to the UHI phenomenon.

Previous studies have shown that air pollution becomes a risk factor for expressing indoor allergen exposure and allergic disease in humans. In particular, the quality air in the surrounding has a significant effect on the severity of asthma (8, 33, 34). In the case of AD, there are fewer related studies than allergic rhinitis or asthma, but there are some reports that suggest that higher HDM levels worsen AD severity (9, 10). However, one of the studies showed these results only in patients who were not sensitized to HDM and were not related to patients that were sensitized (10). In another study, the AD was severe in a group with high *Der p 1* and animal dander allergen, but no effect was found by *Der p 1* concentration alone (9). There was no significant association when comparing PVAS and CADESI in our results, and there similarities with the human reports. Comparing the human studies and our results, it was not related to AD severity when comparing *Der f 1* concentration alone in dogs. However, it is expected that there will be complex associations such as relationships between other allergens in the dust. In addition, HDM sensitization through the intradermal test or specific-serum IgE was not analyzed in this study. In future studies, clearer results could be obtained by adding other allergen in the dust and HDM sensitization to the analysis.

TEWL is an indicator that reflects the amount of the moisture evaporating through extracellular substrates. It is affected by the skin permeability which is determined by the action of keratinocytes and lipids filling between them and is accepted as an indicator of skin barrier function (14). In our results, TEWL and *Der f 1* had a negative correlation in the CAD group. That meant the higher the indoor *Der f 1* concentration, the more substantial the skin barrier function in the CAD group. These results can be related to the high *Der f 1* concentration in the group living near the green area in our study. Previous studies investigated low RH affects skin barrier function and durability in humans and mice (35–39). However, the studies never analyzed the correlation between RH and AD in dogs before.

Considering that hydrating the skin method alleviates AD symptoms in dogs, and the fact that dry skin is the main symptom of CAD (40, 41), RH is expected to affect CAD in humans. Additionally, the inverse correlation between endotoxin and AD was previously reported in humans and dogs (11, 42). It appeared that the lipopolysaccharide (LPS) dose is a determining factor in the course of allergic responses. Low doses of inhaled LPS promoted T helper 2 cell responses to the sensitizing antigen and eosinophilic inflammation, whereas high doses of LPS induced protective T helper 1 cell responses by downregulation of Th2-type cytokines, preventing eosinophilic inflammation, and airway hyperresponsiveness by induction of neutrophil recruitment (43, 44). Endotoxin or bacterial LPS is a component of HDM known to mediate proinflammatory responses *via* the Toll-like receptor innate immune signaling pathway and correlated with asthma risk in humans (45). HDM has a microbiome comprising several bacteria and fungi, and it is expected that HDM extracts contain endotoxins (46). Therefore, a high HDM concentration suggests that the dust has a high level of endotoxin. Although the concentration of endotoxin in the dust was not measured in this study, it is presumed that the related mechanism influenced the TEWL values.

Since the *Der f 1* concentration on average was higher in the CAD group, it could be concluded that the *Der f 1* concentration was independent of CAD occurrence. Further studies through more CAD and control groups will be needed to reveal the association with the occurrence of CAD. In addition, immune sensitization to HDM analysis using neither intradermal test (IDT) nor serum IgE was not performed in this study. It is expected that more significant results could be derived if immune sensitization and *Der f 1* concentration are analyzed. In humans, a cut-off value in which *Der f 1* concentration can cause an immune response has been studied at 2 μg/g (17), but it has never been evaluated in dogs. If the cut-off value is established in future studies, IDT or serum IgE concentration evaluation and analysis of RH are performed, it will be possible to set the optimized RH value to reduce *Der f 1* concentration and symptoms in CAD patients.

To the best of our knowledge, this is the first study to analyze the relationship between AD severity, skin barrier function, and indoor HDM concentration in dogs. It has been revealed that urban green areas can affect indoor *Der f 1* concentration and skin barrier function. RH, as one of the factors that can increase *Der f 1* concentration, can lower TEWL, so it can be suggested that focusing on environmental changes can increase the RH of the living environment in CAD patients rather than *Der f 1* concentration itself will help alleviate patient symptoms. It also suggests the possibility that HDM containing LPS prevent worsening atopic symptoms when presented in high doses. The study may contribute to the important issues regarding AD in veterinary and human medicine, because our study suggests the potential of dogs as a spontaneous disease model for indoor environmental-related AD under the concept of “One Health.”

## 5. Conclusion

This study is the first to analyze the relationship between AD severity, skin barrier function, and indoor HDM concentration in dogs. It has been revealed that urban green areas can affect indoor *Derf1* concentration and skin barrier function. Indoor RH above 40% may play a more important role in alleviating CAD signs than indoor *Der f 1* concentrations.

## Data availability statement

The original contributions presented in the study are included in the article/supplementary material, further inquiries can be directed to the corresponding author.

## Ethics statement

The animal study was reviewed and approved by the Animal Ethics Committee of Chonnam National University and the Institutional Review Board of Chonnam National University (authorization nos. 2021–163 and 1040198-220228-HR-017-01). Written informed consent was obtained from the owners for the participation of their animals in this study.

## Author contributions

JK and J-HL investigated the study. JK acquired and analyzed the data and wrote the manuscript mainly. H-JK and YS designed the study, acquired the fund, edited the manuscript, and supervised the study. All authors contributed to the article and approved the submitted version.
